# Naturally Acquired Human *Plasmodium cynomolgi* and *P. knowlesi* Infections, Malaysian Borneo

**DOI:** 10.3201/eid2608.200343

**Published:** 2020-08

**Authors:** Thamayanthi Nada Raja, Ting Huey Hu, Khamisah Abdul Kadir, Dayang Shuaisah Awang Mohamad, Nawal Rosli, Lolita Lin Wong, King Ching Hii, Paul Cliff Simon Divis, Balbir Singh

**Affiliations:** Malaria Research Centre, Universiti Malaysia Sarawak, Kota Samarahan, Malaysia (T. Nada Raja, T.H. Hu, K.A. Kadir, D.S.A. Mohamad, N. Rosli, L.L. Wong, P.C.S. Divis, B. Singh);; Kapit Hospital, Kapit, Malaysia (K.C. Hii)

**Keywords:** Malaria, zoonoses, *Plasmodium cynomolgi*, *Plasmodium knowlesi*, parasitic diseases, Malaysian Borneo, parasites

## Abstract

To monitor the incidence of *Plasmodium knowlesi* infections and determine whether other simian malaria parasites are being transmitted to humans, we examined 1,047 blood samples from patients with malaria at Kapit Hospital in Kapit, Malaysia, during June 24, 2013–December 31, 2017. Using nested PCR assays, we found 845 (80.6%) patients had either *P. knowlesi* monoinfection (n = 815) or co-infection with other *Plasmodium* species (n = 30). We noted the annual number of these zoonotic infections increased greatly in 2017 (n = 284). We identified 6 patients, 17–65 years of age, with *P. cynomolgi* and *P. knowlesi* co-infections*,* confirmed by phylogenetic analyses of the *Plasmodium* cytochrome c oxidase subunit 1 gene sequences. *P. knowlesi* continues to be a public health concern in the Kapit Division of Sarawak, Malaysian Borneo. In addition, another simian malaria parasite, *P. cynomolgi,* also is an emerging cause of malaria in humans.

*Plasmodium* spp. were identified in the late 1800s, and >30 species have been described in primates, including humans, apes, and monkeys (*1*,*2*). Of these, humans are natural hosts to 4 species: *P. falciparum*, *P. malariae*, *P. vivax*, and *P. ovale.* Human infections with simian malaria parasites were thought to be extremely rare until *P. knowlesi* was identified as a major cause of malaria in humans in Kapit, Malaysian Borneo (*3*). Subsequent human cases of infection with *P. knowlesi* have been reported across Southeast Asia (*4*–*9*). Most cases are reported in the Malaysian Borneo states of Sarawak and Sabah (*4*,*10*,*11*). The zoonotic capability of this parasite was confirmed with the aid of molecular techniques because *P. knowlesi* is morphologically similar to *P. malariae* (*12*). Molecular detection methods also were used to identify other zoonotic malaria parasites infecting humans, such as *P. simium* (*13*,*14*) and *P. brasilianum* (*15*) in South America and *P. cynomolgi* (*16*,*17*) in Southeast Asia.

After the large focus on human *P. knowlesi* infections in the Kapit Division of Sarawak state in 2004 (*3*), extended studies on wild long-tailed macaques (*Macaca fascicularis*) and pig-tailed macaques (*M. nemestrina)* in the area found these species harbor 6 simian malaria parasites: *P. inui*, *P. knowlesi*, *P. cynomolgi*, *P. coatneyi*, *P. fieldi*, and *P. simiovale* (*12*,*18*). Among 108 macaques examined, *P. inui* was the most prevalent parasite (82%), along with *P. knowlesi* (78%) and *P. cynomolgi* (56%). Besides *P. knowlesi*, 2 other simian parasites, *P. inui* and *P. cynomolgi*, have zoonotic capabilities that have been proven through accidental and experimental infections (*1*,*19*–*21*). *P. inui* was experimentally reported to infect humans in 1938, with a subsequent report in 1966 (*20*,*21*). *P. cynomolgi* was reported to infect humans during an accidental transmission in a laboratory in the United States in 1956 and later by experimental trials (*1*,*19*). A single infection of naturally acquired *P. cynomolgi* in a human was described in Peninsular Malaysia in 2014 (*16*) and was confirmed through molecular characterization.

Successful transmission of zoonotic malaria is highly dependent on the bionomics and distribution of competent vectors (*22*). After the 2004 report on human *P. knowlesi* cases in Kapit, *Anopheles latens* mosquitoes were incriminated as the only vector for *P. knowlesi* in the area and were found to harbor sporozoites of other species of simian malaria parasites (*23*). Because wild macaques in Kapit harbored potentially zoonotic malaria parasites and vectors were transmitting *P. knowlesi* to humans, we aimed to determine whether human infections with *P. cynomolgi* and *P. inui* also occurred in the Kapit Division of Sarawak, Malaysian Borneo.

## Materials and Methods

### Study Area, Participants, and Detection of *Plasmodium* spp. 

**Figure 1:**
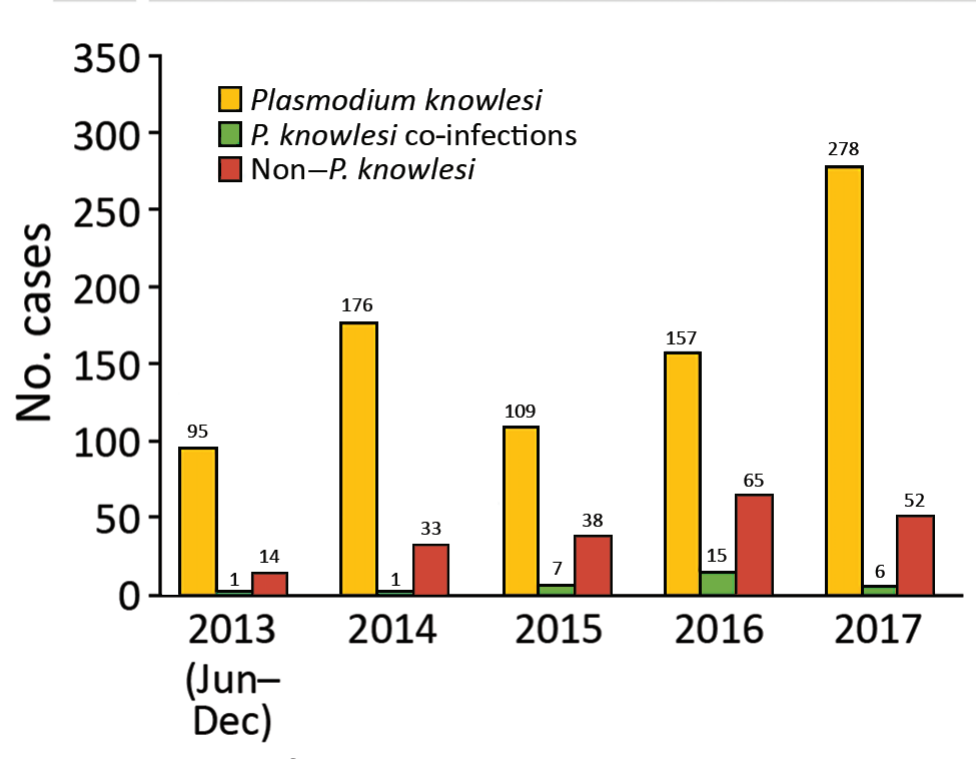
Number of patients admitted to Kapit Hospital with malaria during June 24, 2013–December 31, 2017, Malaysian Borneo. Non–*P. knowlesi* includes *P. falciparum*, *P. malariae*, *P. ovale*, and *P. vivax*. Infections with *Plasmodium* spp. other than *P. knowlesi* each year included the following. In 2013, *P. knowlesi* coinfections included 1 *P. cynomolgi* co-infection; non–*P. knowlesi* included 9 *P. falciparum*, 4 *P. vivax*, and 1 *P. ovale*. In 2014, *P. knowlesi* mixed included 1 *P. falciparum* coinfection; non–*P. knowlesi* included 14 *P. falciparum*, 4 *P. malariae*, 12 *P. vivax*, 3 *P. ovale*. In 2015, *P. knowlesi* co-infections included 3 *P. falciparum* and 4 *P. vivax* co-infections; non–*P. knowlesi* included 16 *P. falciparum*, 16 *P. vivax*, 1 *P. malariae*, 3 *P. falciparum*/*P. vivax* co-infections, 1 *P. falciparum*/*P. ovale* co-infection, and 1 *P. vivax*/*P. ovale* co-infection. In 2016, *P. knowlesi* co-infections included 8 *P. falciparum*, 6 *P. vivax*, and 1 *P. cynomolgi* co-infections; non–*P. knowlesi* included 41 *P. falciparum*, 14 *P. vivax*, 1 *P. ovale*, 3 *P. falciparum*/*P. vivax* co-infections, 1 *P. falciparum*/*P. malariae* co-infection, 4 *P. falciparum*/*P. ovale* co-infections, and 1 *P. vivax*/*P. ovale* co-infection. In 2017, *P. knowlesi* co-infections included 4 *P. cynomolgi*, and 2 *P. vivax* co-infections; non–*P. knowlesi* included 26 *P. falciparum*, 19 *P. vivax*, 3 *P. malariae*, 2 *P. ovale*, 1 *P. falciparum*/*P. vivax* co-infection, and 1 *P. falciparum*/*P. malariae* co-infection.

Kapit is the largest administrative division in Sarawak and has an area of 38,934 km^2^. Most residents in Kapit belong to indigenous ethnic groups living in longhouses close to the forests. Inhabitants who are ill travel to Kapit Hospital for diagnosis and treatment. We enrolled 1,047 patients who had malaria from the Kapit and Song districts who were admitted to Kapit Hospital during June 24, 2013–December 31, 2017 (Figure 1).

Malaria was diagnosed at Kapit Hospital by using blood film examination. Approximately 2 mL of venous blood was collected from each patient. Blood spots on filter paper and thick and thin films were prepared, and 500 µL aliquots of blood were stored frozen. We received samples at the Malaria Research Centre, Universiti Malaysia Sarawak, for further analyses. We extracted *Plasmodium* DNA from dried blood spots by using Instagene Matrix (BioRad Laboratories, https://www.bio-rad.com). We used nested PCR assays to test for the presence of different *Plasmodium* species by using primers specific for *P. falciparum*, *P. vivax*, *P. malariae*, *P. ovale*, *P. knowlesi*, *P. inui*, *P. cynomolgi*, *P. fieldi*, and *P. coatneyi* as described previously (*3*,*24*). We extracted genomic DNA from frozen blood samples by using the QIAamp DNA Mini Kit (QIAGEN, https://www.qiagen.com) and used it for molecular characterization of malaria parasites.

We obtained written consent from all enrolled patients or their parents or guardians for patients <17 years of age. Ethics clearance for this study was obtained from the medical research ethics committee of Universiti Malaysia Sarawak (approval nos. UNIMAS/TNC(AA)-03.02/06-Jld.2[24] and UNIMAS/NC-21.02/03-02 Jld.2[19]) and from the medical research and ethics committee of the Ministry of Health Malaysia (approval nos. NMRR-12-1086-13607[IIR] and NMRR-16-943-31224[IIR]). 

### PCR Amplification and Sequencing of Partial Cytochrome c Oxidase Subunit 1 Gene

Sequencing of the partial cytochrome c oxidase subunit 1 (COXI) gene of *P. cynomolgi* involved a 3-step PCR rather than the 2-step PCR used for *P. knowlesi*. First, we amplified the complete mitochondrial genome by PCR using *Plasmodium*-specific primers Pkmt F1 and Pkmt R1 (*24*). We performed PCR amplification for each sample in a 25-µL reaction mixture containing 1× PCR buffer for KOD FX Neo, 0.4 mmol dNTP mix (Toyobo, https://www.toyobo-global.com), 0.3 µmol of each primer (Pkmt F1 and Pkmt R1), 1 U/25 µL KOD FX Neo polymerase (Toyobo), and 3 µL of purified genomic DNA under the following conditions: 98°C for 30 s for first denaturation; 35 cycles at 98°C for 30 s, 55°C for 30 s, and 72°C for 5 min; then a final extension for 10 min at 72°C. We used S.N.A.P UV Gel Purification Kit (Invitrogen, https://www.thermofisher.com) to perform gel purification of the PCR amplified mitochondrial DNA (mtDNA) whole genome.

We then used the purified mtDNA amplicons as a template for the heminested touchdown PCR (TD-PCR) with *P. cynomolgi*-specific primers: cox1_F1 (5′-CCAAGCCTCACTTATTGTTAAT-3′), cox1_R1 (5′-ACCAAATAAAGTCATTGTTGATCC-3′), and cox1_R3 (5′-ATGGAAATGAGCAATTACATAG-3′). The heminested 1 (N1) TD-PCR amplification for each sample was performed in an 11-µL reaction mixture containing 1× colorless PCR buffer, 2 mmol MgCl_2_, 0.2 mmol dNTP mix, 0.25 µmol of each primer (cox1_F1 and cox1_R1), 0.275 U GoTaq G2 Flexi DNA Polymerase (Promega, https://www.promega.com), and 0.5 µL of purified mtDNA template under the following conditions: 94°C for 3 min for first denaturation; 10 cycles at 94°C for 30 s, 62°C for 45 s, and 72°C for 85 s; 25 cycles at 94°C for 30 s, 53°C for 45 s, and 72°C for 85 s; then a final extension for 5 min at 72°C. After N1 TD-PCR, we performed a heminested 2 (N2) TD-PCR amplification for each of the PCR products in a 22 µL reaction mixture containing similar concentration of PCR master-mix recipe with cox1_F1 and cox1_R1 primers and 1 µL of purified mtDNA template under the following conditions: 94°C for 3 min for first denaturation, 10 cycles at 94°C for 30 s, 59°C for 45 s, and 72°C for 85 s; 25 cycles at 94°C for 30 s, 53°C for 45 s, and 72°C for 85 s; then a final extension for 5 min at 72°C. We used the S.N.A.P UV Gel Purification Kit (Invitrogen) to gel purify the amplicon.

We performed TD-PCR amplification of the *P. knowlesi* COXI fragment by using *P. knowlesi*–specific primers: cox1_Pk_F1 (5′-CATATCCAAGCCTCATTTATGA-3′) and cox1_Pk_R1 (5′-GTGAAAATGAGCAATTACATAA-3′). Amplification was performed in a 22 µL reaction mixture containing similar concentration of PCR master-mix recipe with cox1_F1 and cox1_R1 primers and 1 µL of purified mtDNA template. We used the following thermal cycling parameters: 94°C for 3 min for first denaturation; 10 cycles at 94°C for 30 s, 62°C for 45 s, and 72°C for 85 s; 25 cycles at 94°C for 30 s, 56°C for 45 s, and 72°C for 85 s; then a final extension for 5 min at 72°C. We purified the amplicon as we mentioned in the previous step.

We cloned the purified COXI fragments into TOPO TA Cloning Kit with pCR2.1-TOPO vector (Invitrogen) according to the manufacturer’s protocol. We purified the recombinant plasmids containing the COXI fragment by using the S.N.A.P. Plasmid DNA MiniPrep Kit (Invitrogen) and sent these to a commercial facility for DNA sequencing. For all samples, >3 clones each for *P. cynomolgi* and *P. knowlesi* were sequenced and both forward and reverse strands were sequenced for each clone.

### Computational Analyses of COXI of *Plasmodium* spp.

We used ClustalX version 2 to align the COXI sequences (*25*). We inferred phylogenetic relationships by using the neighbor-joining method in MEGA7 (*26*) then by Bayesian Markov chain Monte Carlo (MCMC) method in the BEAST package version 1.7.5 (*27*). We confirmed the convergence of the chain by inspecting the MCMC samples using Tracer version 1.5 (https://tracer-1-5.software.informer.com), discarded the first 10% sampling of the MCMC chains as burn-in, and conformed the sample size to >200 for all continuous parameters. We used Tree Annotator to annotate the tree generated by BEAST and visualized the maximum clade credibility tree by using FigTree version 1.3.1 (https://figtree-1-3-1.software.informer.com). We deposited *Plasmodium* COXI sequences generated in this study in GenBank under accession nos. MN372324–61 (Appendix).

### Microscopy

We stained thick and thin blood films with Giemsa by using pH 7.2 buffer and examined films under an Olympus BX53 microscope (Olympus, https://www.olympus-lifescience.com). We estimated parasitemia by examining thick blood films and counting parasites associated with <500 leukocytes by 2 independent microscopists. We converted the parasite count to a count per microliter of blood by using the actual leukocyte count of each patient measured with a Sysmex KX-21N Kobe hematology analyzer (Sysmex Corporation, https://www.sysmex.com). We used a light microscope to observe the morphologic characteristics of the parasites in thin blood films.

## Results

### Patient Demographics and Identification of Malaria Parasites 

Of the 1,047 patients, 845 (80.7%) had *P. knowlesi* monoinfections or co-infections with other species of *Plasmodium*, based on nested PCR assays (Figure 1; Table 1). Most (730; 86.3%) patients were >20 years of age, 91 (11%) were 12–19 years of age, and 24 (3%) were <12 years of age; 540 (51.5%) were female and 507 (48.5%) were male. Six patients (codes K07, KT46, K199, K221, K222, and K229) had co-infections with *P. knowlesi* and *P. cynomolgi* (Table 2) without prior history of malaria, and Kapit Hospital initially diagnosed *P. knowlesi* by microscopy. Throughout the 5-year study, we observed a remarkable increase in *Plasmodium knowlesi* malaria cases. In 2017 alone, 284 cases were identified, >2 times that of the highest annual number of cases, 122, admitted during a previous clinical study at Kapit Hospital during 2006–2008 (*28*).

**Table 1:** Comparison of microscopy and PCR for identifying *Plasmodia* spp. among patients with malaria during June 24, 2013–December 31, 2017, Kapit Hospital, Kapit, Malaysian Borneo*

Microscopy	PCR												Total
	Pf	Pk	Pm	Pv	Po	Pf + Pv	Pf + Pm	Pf + Po	Pk + Pf	Pk + Pv	Pk + Pcy	Pv + Po	
*P. falciparum*	93	4	0	2	0	3	0	1	0	0	0	0	103
*P. knowlesi*	7	810	1	12	2	2	1	1	12	11	6	0	865
*P. malariae*	6	1	7	2	0	1	1	1	0	0	0	0	19
*P. ovale*	0	0	0	1	2	0	0	1	0	0	0	1	5
*P. vivax*	0	0	0	48	3	1	0	1	0	1	0	1	55
Total	106	815	8	65	7	7	2	5	12	12	6	2	1,047

* Pcy, *Plasmodium cynomolgi*; Pf, *P. falciparum*; Pk, *P. knowlesi*; Pm, *P. malariae*; Po, *P. ovale*; Pv, *P. vivax*.

**Table 2:** Parasitemia and characteristics of patients with diagnosed *Plasmodium* *cynomolgi and P. knowlesi* coinfections, Malaysian Borneo

Case no.	Year detected	Age, y/sex	Parasites/µL blood
K222	2017	36/F	213
KT46	2013	18/M	334
K229	2017	63/F	1,386
K221	2017	50/F	13,661
K199	2017	17/M	17,072
K07	2016	65/M	84,299

Despite the high number of *P. knowlesi* malaria cases, 19% (202) of malaria cases in Kapit Hospital were infected by human *Plasmodium* species (non–simian *Plasmodium* parasites). During 2013–2016, 2–3 indigenous infections were reported annually, but no indigenous infections were recorded in 2017. Based on travel history, 95% (192/202) of patients infected with nonsimian *Plasmodium* species were timber camp workers who had returned to Sarawak after working in Africa, Papua New Guinea, Brazil, and the Solomon Islands.

### Molecular Characterization of *P. cynomolgi* and *P. knowlesi* Coinfections

**Figure 2:**
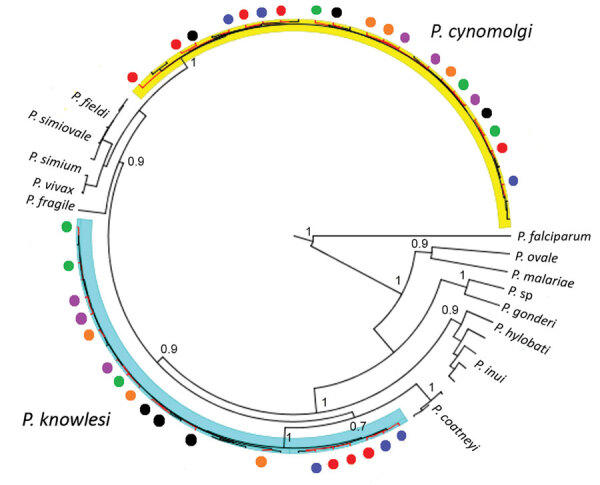
Maximum clade credibility tree for *Plasmodium* cytochrome c oxidase subunit 1 (COXI) sequences from samples from patients admitted to Kapit Hospital with malaria during June 24, 2013–December 31, 2017, Malaysian Borneo. Tree was generated by using strict clock model and Bayesian skyline coalescent tree prior. Circles indicate COXI sequences derived from patients: red indicates patient KT46; black indicates patient K07; orange indicates patient K199; purple indicates patient K221; blue indicates patient K222; and green indicates patient K229.

To confirm the *P. cynomolgi* and *P. knowlesi* coinfections, we obtained *Plasmodium* DNA sequences of 38 partial COXI genes (1,088–1,144 bp) from the 6 patients. The Bayesian phylogenetic inference of these DNA sequences showed 20 sequences formed a monophyletic clade with *P. cynomolgi* referral sequences and was distinct from the *P. vivax* clade. The remaining 18 sequences, however, formed 2 distinct subclades with the *P. knowlesi* referral sequences (Figure 2), confirming the presence of *P. knowlesi* and *P. cynomolgi* parasites in these patients.

### Morphological Characterization of *P. cynomolgi* and *P. knowlesi* Co-infections

**Figure 3:**
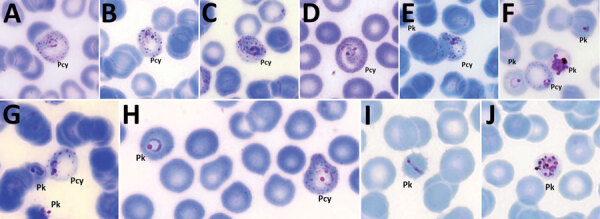
*Plasmodium cynomolgi* and *P. knowlesi* parasites in patient K07, admitted to Kapit Hospital, Kapit, Malaysia, with malaria in 2016. A–G) Early trophozoites of *P. cynomolgi* in enlarged and, at times, distorted erythrocytes, with Schüffner’s stippling and either single, double, or triple chromatin dots. E–H) *P. knowlesi* and *P. cynomolgi* early trophozoites. I) Band form trophozoite of *P. knowlesi*. J) Schizont of *P. knowlesi*. Pcy, *P. cynomolgi*; Pk, *P. knowlesi*. Original magnification ×100.

**Figure 4:**
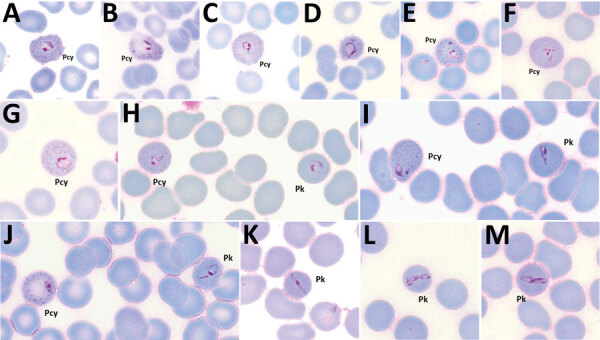
*Plasmodium cynomolgi* and *P. knowlesi* parasites in patient K199, admitted to Kapit , Kapit, Malaysia, with malaria in 2017. A–G) Early trophozoites of *P. cynomolgi* within enlarged and, at times, distorted erythrocytes, with Schüffner’s stippling and single, double, or triple chromatin dots. H–J) Early trophozoites of *P. knowlesi* and *P. cynomolgi*. K–M) Band form trophozoites of *P. knowlesi*. Pcy, *P. cynomolgi*; Pk, *P. knowlesi*. Magnification ×100.

**Figure 5:**
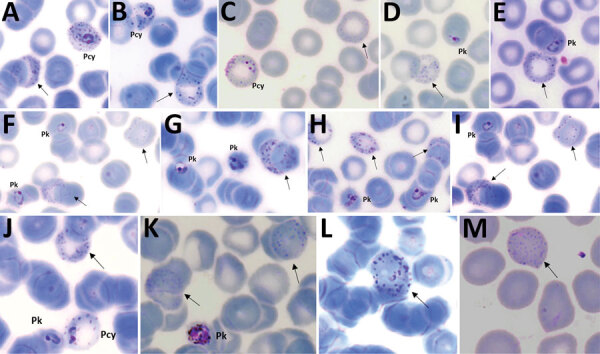
*Plasmodium cynomolgi* and *P. knowlesi* parasites in patient K07, admitted to Kapit , Kapit, Malaysia, with malaria in 2016. Arrows indicate Schüffner’s stippling in erythrocytes without parasites. A–C) Early trophozoites of *P. cynomolgi*; D–I) early trophozoites of *P. knowlesi;* J) early trophozoites of *P. knowlesi* and *P. cynomolgi*; K) gametocyte of *P. knowlesi*. Pcy, *P. cynomolgi*; Pk, *P. knowlesi*. Magnification ×100.

Parasitemia in the 6 patients with *P. cynomolgi* and *P. knowlesi* co-infections ranged from 213 to 84,299 parasites/μL blood (Table 2). Only patients K07 and K199 had blood films of high enough quality for morphologic characterization of *Plasmodium* species. On examination, we noted *P. cynomolgi*–infected erythrocytes constituted a small proportion of the malaria-infected erythrocytes, consisting of ≈1.5% of infected erythrocytes for patients K07 and 4.7% for patient K199. We estimated the percentage of *P. cynomolgi*–infected erythrocytes by using the number of early trophozoite stages with Schüffner’s stippling because we only observed the trophozoite stage for *P. cynomolgi* in these 2 patients. We distinguished *P. cynomolgi* from *P. knowlesi* parasites based on morphologic characteristics of infected erythrocytes, as previously described (*1*). We observed Schüffner’s stippling in early trophozoite-infected erythrocytes, which were enlarged and distorted at times (Figures 3,4). The trophozoites of *P. cynomolgi* also had either single, double, or triple chromatin dots. The growing trophozoites of *P. knowlesi* did not cause erythrocyte enlargement and we did not observe Schüffner’s stippling (Figure 4, panels K, L). Furthermore, we observed band-form trophozoites of *P. knowlesi* in certain erythrocytes (Figure 3, panel I; Figure 4, panel M), and we noted 16 merozoites in 1 schizont (Figure 3, panel J). For patient K07, we examined 4,154 erythrocytes; 12 (0.3%) had prominent Schüffner’s stippling, but we did not observe malaria parasites within these erythrocytes (Figure 5).

## Discussion

The increasing number of patients admitted to Kapit Hospital with *P. knowlesi* infections represents a true increase in infections, not increased awareness, because we used nested PCR to examine *Plasmodium* DNA from blood spots on filter paper from all patients during the study period. The increasing incidence of *P. knowlesi* in the Kapit Division, particularly in 2017; the few patients infected with human malaria parasites, *P. falciparum, P. vivax, P. ovale*, and *P. malariae*, during June 2013–December 2016; and the lack of indigenous cases in Kapit in 2017 reflect the recent malaria situation in Malaysian Borneo and Peninsular Malaysia (*11*). The Ministry of Health, Malaysia, reported 7,745 cases of *P. knowlesi* malaria in Malaysia in 2017 and 2018, and 87% (6,743) occurred in the Malaysian Borneo states of Sarawak and Sabah (*29*). In 2017, only 85 cases of indigenous malaria caused by the human malaria parasites, *P. falciparum, P. vivax, P. ovale*, or *P. malariae* were reported, and none were reported in 2018 (*30*). 

The increase in *P. knowlesi* malaria cases, particularly in Malaysian Borneo, is multifactorial and driven partly by anthropogenic land-use factors leading to changes in the transmission pattern of the parasite between humans, vectors, and macaque reservoirs (*31*). Both states in Malaysian Borneo have undergone substantial deforestation, primarily for palm oil plantations; the total area used for palm oil plantations in Sarawak and Sabah in 2018 was 3.12 million hectares (*32*) compared with just 2.3 million hectares in 2010 (*33*). Such extensive forest clearing activities result in loss of natural habitat for macaques and could have caused them to move closer to human settlements. In Sabah, an association between deforestation and increased incidence of human cases of *P. knowlesi* malaria has been demonstrated (*34*). Furthermore, predictive spatial scale analysis of data from a case-controlled study, a cross-sectional survey, and satellite imagery showed that landscape fragmentation influences *P. knowlesi* transmission to humans (*35*). However, similar studies need to be undertaken in Sarawak to determine whether a direct relationship between land change use and number of *P. knowlesi* malaria cases exists because the vectors in Sarawak are different from those in Sabah. 

Entomologic studies also need to be undertaken because vector bionomics is influenced by landscape factors, and abrupt land use conversion or modifications can change vector distribution, invasion, and behavior (*36*,*37*). One such study conducted in the Kinabatangan area of Sabah in Malaysian Borneo demonstrated that, in a 2-year period *Anopheles donaldi* mosquitoes displaced *An. balabacensis* mosquitoes, the previously dominant vector in that area (*38*). Changes in host preference, biting behavior, and adaptation of the mosquitoes to habitat changes could affect the transmission of zoonotic malaria in Malaysian Borneo. However, determining whether there have been changes in mosquito bionomics in Sarawak is difficult because only 1 study on vector bionomics was conducted in Kapit District in 2004. New detailed entomologic studies at various locations clearly are necessary.

In the 2 patients whose blood films we examined, *P. cynomolgi* parasites comprised ≈1.5% and 4.7% of the total malaria parasites, which explains why microscopy only detected single *P. knowlesi* infections in these patients. The dominance of *P. knowlesi* over *P. cynomolgi* is probably due to the differences in the period of development in the liver and the duration of the erythrocytic cycle between the 2 species. Studies undertaken on the Mulligan (M) strain, and the B strain of *P. cynomolgi* indicated that the incubation period in the liver varied from 15 to 20 days for the B strain and 16 to 37 days for the M strain (*1*), which is much longer than the estimated incubation period for *P. knowlesi* of 9–12 days (*1*). Furthermore, the asexual erythrocytic cycle of *P. knowlesi* is only 24 hours but is 48 hours for *P. cynomolgi* (*1*,*4*). Hence, if *P. knowlesi* and *P. cynomolgi* co-infection occur simultaneously through a single mosquito bite, *P. knowlesi* parasites would emerge from the liver before *P. cynomolgi* and replicate rapidly to become the dominant species infecting the person.

Unlike *P. knowlesi* infections, which can be severe and sometimes fatal (*4*), *P. cynomolgi* infections in humans are mild or even asymptomatic (*1*). In a series of experiments conducted in the United States in the 1960s, volunteers infected with *P. cynomolgi* had mild infections, and for 1 participant the infection persisted for 58 days (*1*,*39*,*40*). A natural infection was reported in a woman with fever in Peninsular Malaysia (*16*), and a tourist from Denmark also had a mild infection with *P. cynomolgi* after returning home from travels in Southeast Asia (*41*). Recently, naturally acquired *P. cynomolgi* and *P. knowlesi* co-infections in Cambodia (*17*) and *P. cynomolgi* monoinfections in Sabah, Malaysian Borneo (*42*), were all asymptomatic. However, our study is hospital-based, so all patients who had *P. cynomolgi* with *P. knowlesi* co-infections were symptomatic.

A recent study described the morphologic characteristics of young to near-mature trophozoites of *P. cynomolgi* in a naturally acquired human host (*41*). We observed only young trophozoites of *P. cynomolgi* in enlarged and at times distorted erythrocytes with Schüffner’s stippling. We observed erythrocytes with stippling without malaria parasites in 1 of the patients. This phenomenon, termed pitting, was initially described for *P. knowlesi* in the 1960s during animal experiments (*43*,*44*). Pitting is a process by which the malaria parasite is expelled from an infected erythrocyte as it passes through the spleen. Once deparasitized through pitting, the erythrocytes return to circulation in the blood. The presence of pitting also was discovered in acute *P. falciparum* infections in which erythrocytes with ring-infected erythrocyte surface antigen and no intracellular parasites in circulation were observed (*45**–**47*). The presence of enlarged deparasitized erythrocytes with Schüffner’s stippling from patient K07 suggests *P. cynomolgi* parasites might have been pitted, leaving the once-infected erythrocytes circulating in the patient’s blood.

The morphologic similarities between *P. cynomolgi* and *P. vivax* have been described previously (*1*,*16*), highlighting the difficulty in correctly identifying *P. cynomolgi* by microscopy. In our study and recent reports of naturally acquired human *P. cynomolgi* infections in Cambodia, Malaysia, and Southeast Asia, molecular methods were necessary to identify *P. cynomolgi* (*17*,*41*,*42*). Microscopy, or rapid diagnostic tests, which are the main methods used in malaria surveys, would have identified the *P. cynomolgi* monoinfections in our study as *P. vivax*. Therefore, the incidence of *P. cynomolgi* in Asia, the natural habitat for several monkey hosts (*1*), is probably much higher than what has been reported so far. Continued surveillance by using molecular methods is needed to obtain accurate data for the incidence of not only *P. cynomolgi*, but also *P. knowlesi*.

In conclusion, we observed that *P. knowlesi* continues to be of public health concern in the Kapit Division of Sarawak, Malaysian Borneo, and that another simian malaria parasite, *P. cynomolgi*, also is an emerging cause of malaria in humans. Further epidemiologic and entomologic studies using molecular tools and a multidisciplinary approach need to be undertaken in Southeast Asia to determine the incidence of *P. cynomolgi* and *P. knowlesi*. Such studies also will assist in determining whether these parasites continue to cause zoonotic infections or whether the loss of natural habitat of macaques, coupled with changes in mosquito composition, abundance, and feeding behavior and an increase in the human population, result in their switch to humans as the preferred hosts.

**Appendix.** Accession numbers of the cytochrome c oxidase subunit 1 gene sequences of *Plasmodium* included in the phylogenetic analysis, including those generated in this study.
